# The Contribution of the Lower Third of the Face to Perceived Age: Do Masks Make You Appear Younger?

**DOI:** 10.1093/asjof/ojab017

**Published:** 2021-05-06

**Authors:** Peter J Nicksic, Alison M Karczewski, Qianqian Zhao, Nicholas A Garcia, Brett F Michelotti, Ashish Y Mahajan, Samuel O Poore

**Affiliations:** Department of Surgery, Division of Plastic Surgery, University of Wisconsin School of Medicine and Public Health, Madison, WI, USA; University of Wisconsin School of Medicine and Public Health, Madison, WI, USA; Department of Biostatistics and Informatics, University of Wisconsin School of Medicine and Public Health, Madison, WI, USA; Department of Surgery, Division of Plastic Surgery, University of Minnesota Medical School, Minneapolis, MN, USA; Department of Surgery, Division of Plastic Surgery, University of Wisconsin School of Medicine and Public Health, Madison, WI, USA; Department of Plastic and Hand Surgery HealthPartners/Regions Hospital, St. Paul, MN, USA; Department of Surgery, Division of Plastic Surgery, University of Wisconsin School of Medicine and Public Health, Madison, WI, USA

## Abstract

**Background:**

There is evidence that changes to the midface and lower third of the face in isolation contribute significantly to one’s perception of the overall facial age. Since the spread of the coronavirus disease 2019 (COVID-19), mask wearing has become commonplace. To date, there have been no studies that explore how covering the lower third of the face impacts the perception of age.

**Objectives:**

The authors hypothesized that covering the lower third of the face with a mask will make a person appear younger.

**Methods:**

One hundred consecutive plastic surgery patients were photographed in a standardized fashion, both masked and unmasked. A questionnaire for factors known to contribute to facial aging was administered. These photographs were randomized to 6 judges who estimated the patients’ age and also quantified facial rhytids with the validated Lemperle wrinkle assessment score of 6. Data were analyzed using PROC MIXED analysis.

**Results:**

Masked patients on average appeared 6.17% younger (mean difference = 3.16 years, *P* < 0.0001). Wrinkle assessment scores were 9.81% lower in the masked group (mean difference = 0.21, *P* = 0.0003). All subgroups appeared younger in a mask except for patients aged 18 to 40 years chronological age (*P* = 0.0617) and patients BMI > 35 (*P* = 0.5084).

**Conclusions:**

The mask group appeared younger and had lower overall and visible wrinkle assessment scores when compared with the unmasked group. This has implications for our understanding of the contributions of the lower third of the face to overall perceived facial age.

In 2018, the cosmetic surgery market generated an estimated 16.5 billion dollars in the United States according to the American Society of Plastic Surgeons.^1^ Much of this revenue is attributable to patients seeking to reduce or reverse facial aging—a well-studied process that alters the appearance of facial features.^1^ Over time, skin elasticity reduces, retaining ligament integrity lessens, and volume of soft tissue and fat compartment shift. These changes together constitute the appearance of advancing facial age.^2^ Environmental factors have been identified to accelerate this process, including smoking, alcohol use, and sun exposure.^3-8^ Interestingly, higher BMI has been shown to be a “normalizing” factor for age, making both younger and older people appear to be middle-aged.^3^ Gender differences and genetic factors such as sex hormones, skeletal anatomy, and facial physiology also play a role in the perception of age.^9^ For example, larger eyes, taller lips, and greater facial contrast are all associated with appearing younger.^10-12^

In addition to these intrinsic and extrinsic factors, the contribution of specific subunits of the face has been shown to disproportionately affect our perception of age. The midface, including the central facial triangle, is shown to be an important determinant of perceived attractiveness and age.^13^ Artificially aging the lower third of the face with computer-based photograph editing significantly increases the perceived age of a person and is among the most determining of the facial subunits.^13^ To date, there have been no studies that have explored how covering subunits of the face with a mask affect the perception of age.

Before 2020, the use of face masks in the United States was largely limited to the healthcare setting. However, as the respiratory virus causing coronavirus disease 2019 (COVID-19) began to spread worldwide with increasing morbidity and mortality, the use of face masks in the public setting became more common as many state governments began issuing mask mandates.^14^ Despite there being evidence to support the use of face masks in preventing disease transmission, it remains a controversial topic driven by political divide, individualism, and distrust of authority.^15-17^

The purpose of our study is to better understand the effects of wearing a mask on the perception of facial age. We hypothesize that based on our own observation, and well-documented and studied contribution of the lower third of the face to our perception of age,^13^ face masks make individuals appear younger. The implications of appearing younger extend beyond physical attractiveness. Looking younger than chronological age is associated with increased trustworthiness, competence, and warmth,^12,18^ whereas looking older correlates with decreased optimism, relationship satisfaction, and mental health.^19^ Even cognitive function has demonstrated a stronger correlation with perceived age than with chronological age.^20^ This study may also provide more insight into how the morphological features of each subunit of the face contribute to the perception of age, and if mask wearing makes one appear more youthful, the results of this study may highlight a benefit of mask wearing.

## METHODS

This study was conducted in accordance with the guidelines set forth in the Declaration of Helsinki. After obtaining IRB approval at The University of Wisconsin, we collected 6 standardized photographs of 100 patients from the clinic of 2 senior authors, B.F.M. and S.O.P., from October 2020 to February 2021. All patients were offered a standardized light blue, around-the-ear surgical mask. Photographs were all taken with the same background and included a right lateral, frontal, and left lateral. The same photographs were taken with and without a mask. Inclusion criteria were any adult patient being seen in the plastic surgery clinic. Exclusion criteria were any patient being seen for facial pathology and those under 18 years of age. We also administered a questionnaire to obtain demographic information and a social history of factors known to contribute to facial aging (Appendix, available online at www.asjopenforum.com).^3^ Questions were asked by members of the research team in a face-to-face manner, while answers were written down on paper. Patients were assured their answers would remain anonymous. Data were collected during a single plastic surgery clinic visit so as to not further increase the patients’ exposure to a healthcare setting amidst the COVID-19 pandemic. Informed consent and photograph release were also obtained at this time. 

The photometric data were then reviewed independently by 6 board-certified plastic surgeons from our institution. The judges were blinded to the goal of the study and were not directly involved in patient care of any of the study participants. Each judge estimated the age and severity of facial wrinkles according to the validated Lemperle wrinkle assessment scale^21^ of either the masked or unmasked photographs for each patient. When a mask concealed a portion of the patient’s face, the wrinkles that were not visible were not scored and excluded from analysis. This was the overall wrinkle assessment score. We also compared wrinkle assessment scores for wrinkles that were visible in all patients, including the forehead, glabella, periorbita, preauricular rhytids, and neck. This was the *visible* wrinkle assessment score. Each judge appraised each patient once, either masked *or* unmasked, but never saw the same patient masked *and* unmasked. The mask status of the patients was randomized to the judges with equal weight by a biostatistician from the Department of Biostatistics and Informatics biostatisticians using R software. Power analysis was performed with assistance from the Department of Biostatistics and Informatics biostatisticians using a paired *t* test based on an anticipated effect size of 19.8% increase on perceived age attributable to artificially aging the lower third of the face from a previous study.^13,22^ A sample size of 199 data pairs achieves 80.0% power to reject the null hypothesis of zero effect size when the population effect size is 0.20 and the significance level (α) is 0.05 using a 2-sided paired *t* test, and 90 data pairs achieves 80.0% power to reject the null hypothesis of zero effect size when the population effect size is 0.30. As such, we collected data on 100 consecutive patients and performed the statistical analysis.

Statistical analyses were conducted using SAS software (SAS Institute Inc., Cary, NC), version 9.4. Descriptive statistics such as mean and standard deviation (SD) were calculated for continuous variables, and count and frequency were generated for categorical variables. To determine if masked patients appeared younger than unmasked patients, the data were analyzed using “PROC MIXED” since each patient was reviewed multiple times by different surgeons. Both the *visible* and *overall* wrinkle assessment scores were compared between the masked and unmasked groups using the same method. Analysis was also performed to determine if the unmasked group was statistically different than the patients’ actual age. Subgroup analyses were carried out based on gender, BMI, chronological age, smoking history, and alcohol intake. We divided the age groups before data collection to represent a young cohort (18-40 years of age), middle-aged cohort (41-65 years of age), and elderly cohort (66 and older). For the purpose of this study, greater than or equal to 2 standard drinks per week qualified a person as an alcohol consumer, and greater than or equal to 5 pack-years smoking history qualified a person as a smoker. Inter-rater reliability was assessed using Shrout-Fleiss’ intraclass correlations (ICCs),^23^ and the judges all rated the quality and consistency of the photographs on a 5-point scale.

## RESULTS

Our study included 100 consecutive patients seen in plastic surgery clinic from October 2020 to February 2021. The average age was 50.75 years (SD = 12.73, range, 24-79), and the average BMI was 29.4 (SD = 8.02, range, 17.1-73.0). Eighty patients were female and 20 were male. Twelve percent of patients were active smokers with an average pack-year history of 4.18 (SD = 11.22, range, 0-80). The average number of alcoholic drinks per week was 2.12 (SD = 2.54, range, 0-12). The percentages of patients with histories of blistering sunburns and skin cancer were 48% and 12%, respectively. Twenty percent of patients used sunscreen daily, 53% used sunscreen for exposure, and 27% did not use sunscreen. Eleven percent of patients had Botox treatment in the past, and 3% had a history of facial filler. Two patients had a facelift in the past, and 3 patients had a brow lift. The demographic data are summarized in Table 1.

**Table 1.** Demographic Data for the Study Population

	Mean (SD)	Median (range)
Age	50.75 (12.73)	49.5 (24-79)
Height (m)	1.67 (0.09)	1.67 (1.47-1.96)
Weight (kg)	82.38 (24.64)	79.5 (42.2-231.3)
BMI	29.37 (8.02)	28.2 (17.1-73.0)
Cigarettes per day	3.97 (7.68)	0 (0-40)
Years smoking	6.20 (11.21)	0 (0-40)
Pack-years	4.18 (11.22)	0 (0-80)
Alcohol drinks per week	2.12 (2.54)	1 (0-12)
	Number	Percent (%)
Female	80	80
Male	20	20
Cigarette smokers (>5 pack-years)	12	12
Face lift	2	2
Botox	11	11
Filler	3	3
Brow lift	3	3
Neck lift	1	1

SD, standard deviation.

Inter-rater reliability was assessed to be Shrout-Fleiss’ ICC = 0.78 and ICC = 0.62 for estimated age and wrinkle scale score, respectively (ICC > 0.60 is considered substantial agreement, and ICC < 0.30 is considered problematic for comparison between judges.). The photograph quality was scored at an average 4.83 out of 5. Consistency between masked and unmasked patients was scored at 4.5 out of 5.

The perceived age for masked patients was determined to be 48.03 years (95% CI = 45.34-50.72). The perceived age for unmasked patients was determined to be 51.19 years (95% CI = 48.50-53.88). Masked patients were determined to be significantly younger in appearance than masked patients by 3.16 years (95% CI = 2.26-4.06, *P* < 0.0001). The overall wrinkle assessment scores for masked patients were 1.85, whereas the masked group were 2.06 (mean difference = 0.21, 95% CI = 0.10-0.32, *P* = 0.0003). Visible wrinkle assessment scores for masked patients were 1.92, and the unmasked group was 2.06 (mean difference = 0.15, 95% CI = 0.04-0.25, *P* = 0.0082). The perceived age of the unmasked group was determined not to be statistically different from the patients’ actual age (mean difference = 0.45, *P* = 0.4344). The results of perceived age and wrinkle assessment score data are summarized in Table 2.

**Table 2.** Perceived Age and Wrinkle Assessment Score Overall and for Each Subgroup Analyzed

Outcome	Subgroup	Unmasked average	95% CI	Masked average	95% CI	Difference average	95% CI	*P*-value
Perceived age	Overall	51.19	(48.50 to 53.88)	48.03	(45.34 to 50.72)	3.16	(2.26 to 4.06)	<0.0001
	Female	51.87	(49.09 to 54.64)	48.46	(45.68 to 51.23)	3.41	(2.36 to 4.46)	<0.0001
	Male	48.47	(40.69 to 56.26)	46.30	(38.52 to 54.08)	2.17	(0.59 to 3.76)	0.0077
	Age (18-40)	33.33	(31.03 to 35.64)	31.58	(29.28 to 33.89)	1.75	(−0.09 to 3.59)	0.0617
	Age (41-65)	51.72	(49.28 to 54.15)	48.26	(45.83 to 50.70)	3.46	(2.23 to 4.68)	<0.0001
	Age (66+)	69.22	(66.24 to 72.21)	65.46	(62.48 to 68.45)	3.76	(1.99 to 5.53)	<0.0001
	Nonsmoker	50.43	(47.38 to 53.47)	47.75	(44.71 to 50.80)	2.68	(1.67 to 3.68)	<0.0001
	Smoker	54.40	(48.65 to 60.16)	49.15	(43.38 to 54.92)	5.25	(3.22 to 7.29)	<0.0001
	BMI < 35	50.70	(47.75 to 53.65)	46.92	(43.97 to 49.86)	3.78	(2.79 to 4.78)	<0.0001
	BMI >= 35	53.15	(46.65 to 59.65)	52.45	(45.95 to 58.95)	0.70	(−1.39 to 2.79)	0.5084
	Female smoker	53.20	(47.02 to 59.38)	47.48	(41.27 to 53.69)	5.72	(3.03 to 8.41)	<0.0001
Overall wrinkle assessment score	Overall	2.06	(1.84 to 2.28)	1.85	(1.63 to 2.07)	0.21	(0.10 to 0.32)	0.0003
	Female	2.07	(1.84 to 2.30)	1.83	(1.60 to 2.06)	0.24	(0.11 to 0.37)	0.0002
	Male	2.03	(1.40 to 2.66)	1.95	(1.32 to 2.58)	0.08	(−0.14 to 0.30)	0.4706
	Age (18-40)	0.70	(0.53 to 0.87)	0.65	(0.48 to 0.82)	0.05	(−0.13 to 0.23)	0.5896
	Age (41-65)	2.11	(1.88 to 2.34)	1.92	(1.69 to 2.15)	0.19	(0.04 to 0.33)	0.0119
	Age (66+)	3.42	(3.07 to 3.77)	2.96	(2.61 to 3.31)	0.46	(0.16 to 0.77)	0.0034
	Nonsmoker	1.99	(1.75 to 2.23)	1.77	(1.53 to 2.01)	0.22	(0.10 to 0.35)	0.0005
	Smoker	2.36	(1.82 to 2.91)	2.22	(1.67 to 2.77)	0.14	(−0.11 to 0.40)	0.2678
	BMI < 35	2.00	(1.76 to 2.23)	1.77	(1.54 to 2.00)	0.23	(0.10 to 0.35)	0.0005
	BMI >= 35	2.33	(1.75 to 2.92)	2.19	(1.60 to 2.77)	0.15	(−0.09 to 0.38)	0.2209
	Female smoker	2.18	(1.58 to 2.77)	2.07	(1.47 to 2.67)	0.10	(−0.24 to 0.44)	0.5468

BMI, body mass index; CI, confidence interval.

Subgroup analyses revealed that females looked 3.41 years younger (95% CI = 2.36-4.46, *P* < 0.0001) when masked, and males looked 2.17 years younger when masked (95% CI = 0.59-3.76, *P* = 0.0077). The overall wrinkle scores were 0.24 lower when masked in the female subgroup (*P* = 0.0002) but not significant in the male subgroup (mean difference = 0.08, *P* = 0.4706). Perceived age (*P* = 0.6183) and wrinkle assessment (*P* = 0.3921) scores were not significant when compared on the basis of gender.

**Figure 1.**
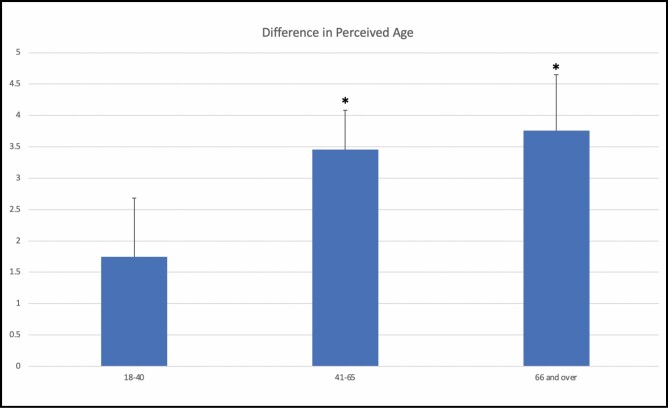
The difference in perceived age by age group. The young age group (18-40 years of age) demonstrated an insignificant mask effect of 1.75 years (*P* = 0.0617). The middle-aged group (41-65 years of age) demonstrated a mask effect of 3.46 years (95% CI = 2.23-4.68, *P* < 0.0001). The elderly group (66 years and older) demonstrated a mask effect of 3.76 years (95% CI = 1.99-5.53, *P* < 0.0001). Significant age groups are denoted by an asterisk..

When subdivided by age groups, the difference in perceived age increased with actual age (Figure 1). In the 18- to 40-year-old subgroup, the mean difference in perceived age was 1.75, which was not significant between the masked and unmasked subgroups (*P* = 0.0617). In the 41- to 65-year-old subgroup, the mean difference was 3.46 years (95% CI = 2.23-4.68, *P* < 0.0001). In the 66 years of age and older subgroup, the mean difference was 3.76 years (95% CI = 1.99-5.53, *P* < 0.0001).

Smokers appeared on average 5.25 years younger when masked (95% CI = 3.22-7.29, *P* < 0.0001), whereas nonsmokers demonstrated a mean difference of 2.68 years (95% CI = 1.67-3.68, *P* < 0.0001). Female smokers appeared 5.72 years younger in the masked group (95% CI = 3.03-8.41, *P* < 0.0001). The average wrinkle scores for smokers were not statistically different when comparing masked and unmasked groups (*P* = 0.2678). Perceived age (*P* = 0.8803) and wrinkle assessment scores (*P* = 0.3735) were not significant for smokers vs nonsmokers. Alcohol consumers were found to have a difference of perceived age of 2.72 years (95% CI = 1.25-4.19, *P* = 0.0003), where judges determined that there was a difference of 3.42 years in nondrinkers (95% CI = 2.27-4.56, *P* < 0.0001). When comparing the differences in perceived age (*P* = 0.9874) and wrinkle assessment scores (*P* = 0.5623) between alcohol consumers and nondrinkers, this was not statistically significant.

Obese patients demonstrated no difference in perceived age (*P* = 0.5084) or wrinkle assessment score (*P* = 0.2209) between the masked and unmasked group. Perceived age (*P* = 0.2368) and wrinkle assessment scores (*P* = 0.1590) were not significant when comparing obese vs nonobese patients.

**Figure 2.**
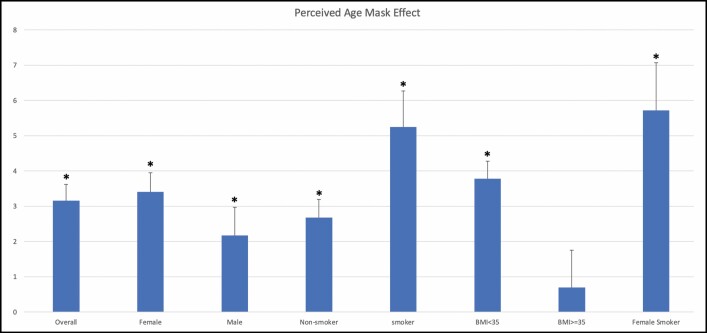
The difference in perceived age overall and for all subgroups. The overall mask effect was 3.16 years (*P* < 0.0001). Female smokers demonstrated the largest mask effect of 5.72 years (*P* < 0.0001). Obese patients demonstrated a nonsignificant mask effect (*P* = 0.51). Significant subgroups are denoted by an asterisk. BMI, body mass index.

**Figure 3.**
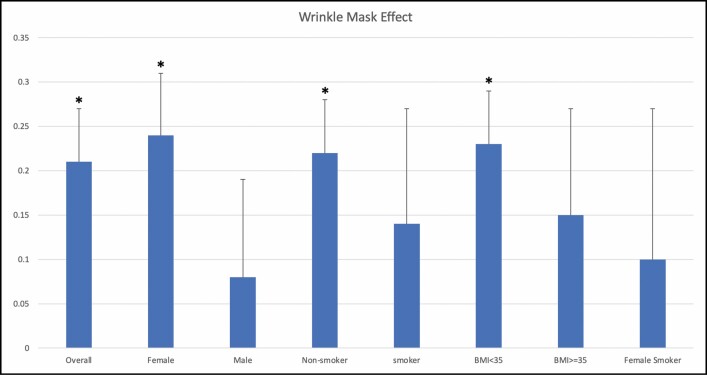
The difference in wrinkle assessment score overall and for all subgroups. The overall mask effect was 10.19% less wrinkles (*P* = 0.0003). Females demonstrated the largest mask effect of 11.67% less wrinkles (*P* < 0.0001). Significant subgroups are denoted by an asterisk. BMI, body mass index.

When controlling for all effect modifiers—gender, age, smoking, alcohol consumption, and BMI—the mask group demonstrated a lower perceived age than the unmasked group (mean difference 3.17, 95% CI = 2.27-4.07, *P* < 0.0001) and lower overall wrinkle score (mean difference 0.2097, 95% CI = 0.10-0.32, *P* = 0.0002). Figures 2, 3 show the average difference in age and winkle score, respectively, for all patients and subgroups.

## DISCUSSION

The “mask effect,” or the effect on perceived age attributable only to mask wearing, made patients appear on average 6.17% younger (mean difference = 3.16 years, 95% CI = 2.26-4.06, *P* < 0.0001). This effect size is similar to other studies that examined the effect of aesthetic surgery on perceived age. A prospective study of 49 facial aesthetic surgery patients demonstrated a reduction in perceived age of 3.1 years from preoperative to postoperative photographs.^24^ Another study of 10 hyaluronic acid injection patients demonstrated a reduction in the age of 6.1 to 7.3 years 2 to 4 weeks after the procedure.^25^ While there is a relatively modest reduction in perceived age attributable to mask effect in this study, the reduction in perceived age is on the same order of magnitude as of other popular facial rejuvenation strategies today.

**Figure 4.**
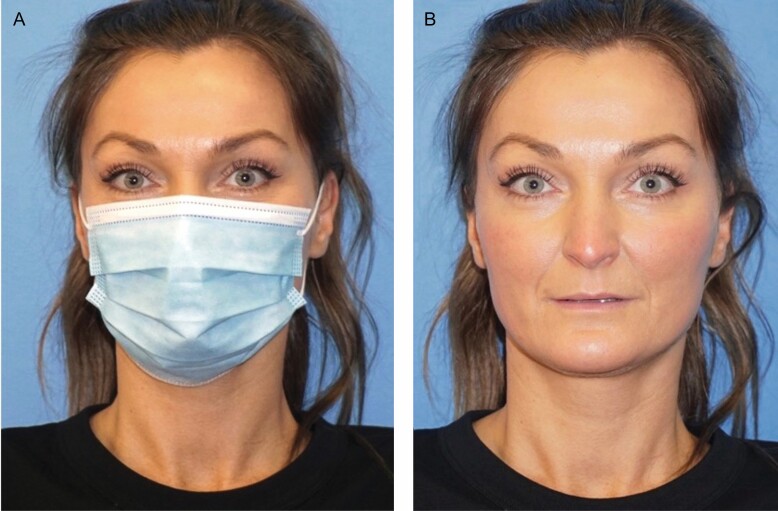
Pictured is a 37-year-old female, body mass index of 20, without a smoking or alcohol history (A, B). The dramatic “mask effect” is evident and is potentially amplified by her history of Botox treatments. Her average masked perceived age was 28.67 years.

One study on the topic of facial subunits and aging found that artificially aging the lower third of the face in isolation made a patient appear 19.8% older (*P* < 0.0001).^13^ While it is logical to assume that obscuring the lower third would have less of an effect on perceived age than artificially aging it, the question still remains: why does wearing a mask affect our perception of age at all? One reason is that the aging lower third of the face contains vertical lip rhytids, nasolabial folds, and jowls, which have been demonstrated to contribute significantly to our perception of facial aging even in isolation.^3^ If a person does not have a significant forehead, glabellar, or periorbital wrinkles, one would infer that the person is overall younger if they are obscuring their lower face with a mask. Unfortunately, too few patients in our study population have had cosmetic surgery or injectables to see if glabellar and forehead Botox increased the mask effect or a previous facelift reduced the mask effect. However, the dramatic effect of previous upper face Botox can be seen in one patient (Figure 4).

According to annual procedural statistics from the American Society of Plastic Surgeons, the number of treatments with botulinum toxin type A increased from 786,911 in the year 2000 to 7,697,798 in 2019—almost a 10-fold difference.^1^ Anecdotally, nonsurgical aesthetic treatments such as Botox and fillers have grown remarkably during the pandemic. A plausible reason for this is the amount of time that many of the patients now spend in virtual meetings where they can see their own faces magnified on a screen, potentially increasing a sense of self-consciousness. The mask effect, understood consciously or unconsciously, is another possible reason.

**Figure 5.**
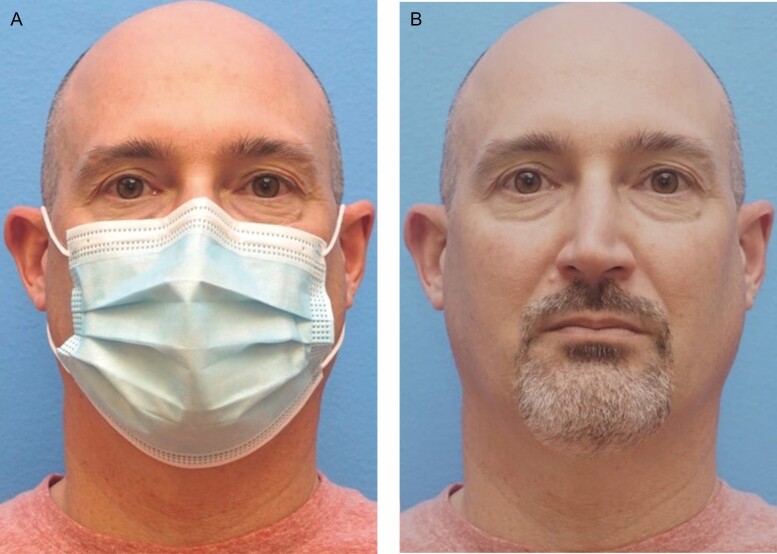
Pictured is a 51-year-old male nonsmoker, body mass index of 28, with 2 drinks per week of alcohol consumption (A, B). The male subgroup “mask effect” is possibly affected by male-pattern baldness. His average masked perceived age was 43.00 years.

While all subgroups—except patients whose BMI was greater than 35 and patients aged 18 to 40—demonstrated a significant mask effect to variable degrees, there was no significant difference between subgroups for either perceived age or wrinkle scores. With a larger study powered to detect differences between subgroups, the variable mask effects may be significant. For example, females demonstrated a mask effect of 3.41 years (95% CI = 2.36-4.46, *P* < 0.0001), which was not significant when compared with the 2.17-year mask effect in males (95% CI = 0.59-3.76, *P* = 0.0077). Our study was not powered to determine the difference in mask effect based on gender. However, women invest more in “anti-aging” products such as skin care products, hair coloring, and make-up.^26^ Male-pattern baldness may also have been a tell of age that was not concealed by a mask (Figure 5). However, degree of baldness was not associated with an overestimation of age in one photometric study.^27^ So, it is possible that a larger study with equivalent numbers of males and females may detect a larger mask effect in females. There was also an insignificant effect for wrinkle assessment score in males (mean difference = 0.08, *P* = 0.4706). This may be due to the fact that subgroup analysis in the study was underpowered to detect this difference, or it may be due to facial hair obscuring lower third facial wrinkles in men.

**Figure 6.**
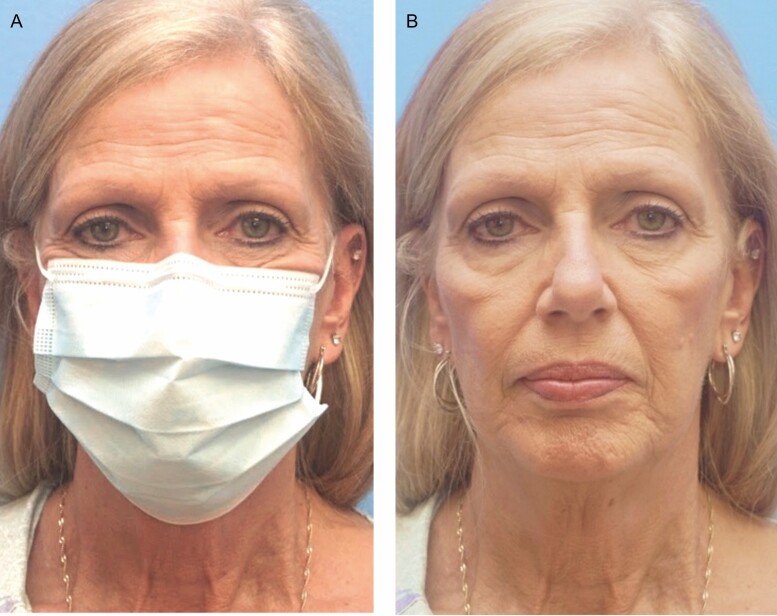
Pictured is a 56-year-old female, body mass index of 24, with a 20 pack-year smoking history (A, B). Note her exaggerated lower face wrinkling, contributing to a significant “mask effect.” Her average masked perceived age was 50.00 years.

Patients with known extrinsic risk factors for premature aging such as smoking and alcohol consumption showed variable effects on perceived age. For smokers, the mask effect was responsible for making them appear 5.25 years younger (95% CI = 3.22-7.29, *P* < 0.0001), whereas their nonsmoking counterparts demonstrated a mask effect of 2.68 years (95% CI = 1.67-3.68, *P* < 0.0001). When compared with nonsmoker identical twins, smokers demonstrated disproportionate premature aging of the lower face, including vertical lip rhytids, jowls, and nasolabial folds.^8^ We hypothesize that future studies would demonstrate an increased mask effect in these patients, and these signs of smoking-related facial aging are evident in our study (Figure 6).

**Figure 7.**
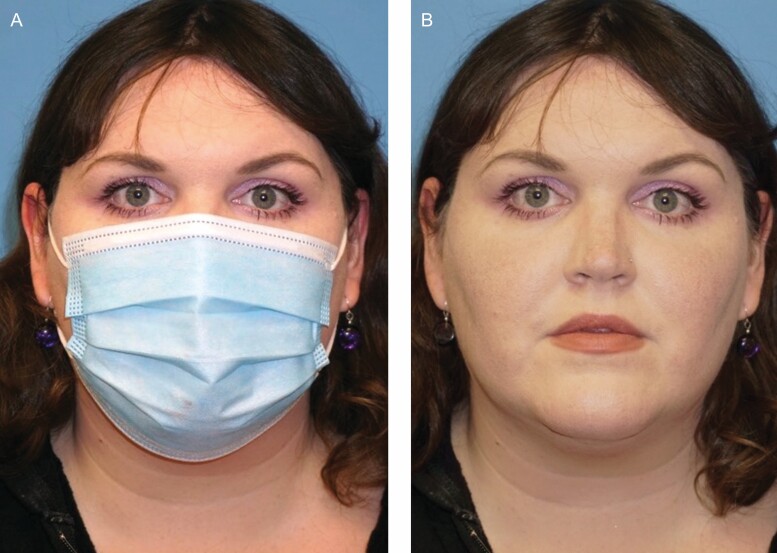
Pictured is a 40-year-old female, body mass index (BMI) of 46, without a smoking or alcohol history (A, B). Subgroup analysis of patients with BMI > 35 showed no significant mask effect (*P* = 0.5084). Her average masked perceived age was 48.00 years.

Interestingly, subgroup analysis for patients with BMI > 35 had insignificant changes to their perceived age and wrinkle assessment scores (*P* = 0.51, 0.22, respectively). In another twin study, a BMI 8 points higher than the respective twin was associated with a higher perceived age in patients under 40 years old and a younger perceived age in patients over 55 years old.^3^ Elevated BMIs are associated with reduced wrinkling in all subunits of the face,^3^ which we hypothesize would reduce the mask effect (Figure 7).

The subgroup with the largest mask effect was in women who smoked and who appeared 5.72 (10.75%) years younger with a mask (95% CI = 3.02-8.41, *P* < 0.0001). This finding indicates that individuals with a history of smoking will significantly benefit from rejuvenation procedures focused on the lower two-thirds of the face.

### Limitations

One interesting effect is that, overall, masked patients were shown to have a lesser visible wrinkle assessment score. The masked individuals demonstrated a 7.09% reduction in wrinkle score (mean difference = 0.1463, 95% CI = 0.03798-0.2547, *P* = 0.0082). One would assume that the *visible* wrinkle assessment score—scoring averaged only for forehead, glabellar, periorbital, preauricular, and neck wrinkles—between the masked and unmasked groups would be equivalent if the photography of the masked and unmasked patients were consistent. Therefore, it is possible that this effect may be due to a subtle flash artifact in masked patients, or it is possible that obscuring more significant lower facial wrinkles reduced the appearance of all wrinkles in the face. The near-perfect average consistency score (4.5 out of 5) for the photographs of masked vs unmasked patients supports that wearing a mask affects one’s perception of the entire face.

In order to minimize potential judgment bias, we made the decision to show judges a mix of mask and unmasked patients without showing a judge the same patient masked and unmasked. The judges were blinded to the goal of the study, but due to the subject matter of the study, it is possible that they inferred the goal, which introduced another potential source of judgment bias.

The power analysis was performed to compare the entire sample masked vs unmasked. This increases our risk of a beta-type error in subgroup analysis. The majority of subgroups—except morbidly obese patients and patients under 40 years of age—demonstrated a reduction in perceived age when masked even with what is likely insufficient power for the anticipated effect.

Our study population consisted of 80% females. Each participant acted as his/her own control, so there were equivalent numbers of males and females in the masked vs unmasked groups. The difference in mask effect of the possible effect modifiers—obesity, smoking, and alcohol consumption—was nonsignificant between subgroups. Therefore, despite there being a disproportionate number of females in the study, the results of the study can still be applied to both genders and do not affect the overall conclusion of the study. The average BMI of the study population was 29.4 and contained 12% smokers. These statistics are similar to the World Health Organization’s estimates for the United States for average BMI (29.1) and prevalence of smokers (14%).

## CONCLUSIONS

This study demonstrates that there is a significant mask effect—that those who wear a mask appear younger on average and have a lower overall and visible wrinkle score. This effect was seen to increase with age but was not significant between subgroups of patients with known risk factors for premature facial aging such as smoking and consuming alcohol. These findings expand our understanding of the importance of the lower third of the face and its role in an observer’s perception of age. When determining what facial subunit to address first in facial rejuvenation, the findings of this study suggest that treating the lower third with surgery or injectables will reduce the patient’s perceived facial age.

Furthermore, the mask effect offers a positive incentive for mask wearing. Given the resistance nationally to mask wearing due to political views and distrust of authority,^15-17^ incentivizing mask wearing may play an integral role in overcoming the COVID-19 pandemic. Similar to national mandates for mask wearing, there has been resistance with other public health initiatives such as helmet and seat belt use. Normalization and positive reinforcement helped encourage compliance and promote new societal norms.^28^ Widespread acceptance of these critical safety measures required perspectives that went beyond scientific data and risk management. Mask wearing highlights an additional component of altering a person’s physical appearance—that is, makes the wearer appear younger.

## Supplementary Material

ojab017_suppl_Supplementary_Materials
