# Supplementary material for: The Contribution of the Lower Third of the Face to Perceived Age: Do Masks Make You Appear Younger?
Source: Aesthet Surg J Open Forum. 2021 May 6;3(3):ojab017. doi: 10.1093/asjof/ojab017 (PMC8241419; doi:10.1093/asjof/ojab017)
Supplement: ojab017_suppl_Supplementary_Materials [file ojab017_suppl_supplementary_materials.pdf]

Mask Study Questionnnaire

Record ID	________________
Participant Number	________________
Age	________________
Gender	◯ Male<br>◯ Female<br>◯ Non-binary<br>◯ Choose not to disclose
Height (in meters)	________________
Weight in kilograms	________________
BMI	________________
Currents Tobacco Use?	☐ Cigarettes<br>☐ Chewing Tobacco<br>☐ Vaping<br>☐ None
Cigarettes per Day	________________
Years Smoking	________________
Alcoholic Drinks Per Week	________________
Personal History of Skin Cancer?	◯ Yes<br>◯ No
History of Blistering Sun Burns?	◯ Yes<br>◯ No
Amount of Sunscreen Use	◯ daily<br>◯ for exposure<br>◯ none
History of Facial Cosmetic Operations	☐ Facelift<br>☐ Botox<br>☐ Filler<br>☐ Brow lift<br>☐ Blepharoplasty<br>☐ Neck lift
